# Fasting before contrast-enhanced CT and the incidence of acute adverse reactions: a single-center randomized clinical trial

**DOI:** 10.1186/s13244-024-01767-9

**Published:** 2024-08-07

**Authors:** Laila Zitan Saidi, Maricela Moreira Cabrera, Teresa Góngora Lencina, Fuensanta Marín Morón, Raquel Alarcón Rodríguez, Jessica García González

**Affiliations:** 1 Department of Diagnostic Imaging, Torrecárdenas University Hospital, Almería, Spain; 2 Department of Diagnostic Imaging, Vall d’Hebron University Hospital, Barcelona, Spain; 3 Department of Nursing, Physiotherapy and Medicine, Faculty of Health Sciences, University of Almería, Almería, Spain

**Keywords:** Fasting, Spiral computed tomography, Contrast media, Nausea

## Abstract

**Objectives:**

To evaluate the effect of eliminating the traditional preparatory fasting policy before contrast-enhanced CT on acute adverse reactions and to identify potential risk factors in a Spanish population sample, since many European patients still experience this unnecessary measure in clinical practice.

**Methods:**

Outpatients who underwent non-emergency CT to either 6 h of solid food fasting (control group) or an unrestricted consumption of solids (intervention group). Adverse reactions during contrast media administration and up to 30 min afterward were recorded and their incidence was calculated. Using univariate and multivariate logistic regression analyses, various patient-related and technical factors were evaluated to identify risk factors for nausea and vomiting.

**Results:**

One thousand one hundred three patients were evaluated, 560 patients in the control group, and 543 patients in the intervention group. Moderate and severe acute adverse reactions were not identified in either group. No statistical difference was found in the overall acute adverse reactions (hypersensitivity and chemotoxicity) incidence between groups (3.21% vs 2.30% *p* = 0.36). The total incidence of emetic adverse reactions (nausea and vomiting) was significantly lower in the intervention group than in the control group (0.92% vs 2.86% *p* = 0.02). Multivariate logistic regression analysis revealed that fasting, age, allergies, neurological diseases, and contrast media concentration were independent risk factors for nausea and vomiting.

**Conclusion:**

Unrestricted food intake did not increase the overall incidence of acute adverse reactions and diminished the incidence of nausea and vomiting.

**Trial registration:**

ANZCTR, ACTRN12623000071628. Registered 23 January 2023—retrospectively registered, https://www.anzctr.org.au/Trial/Registration/TrialReview.aspx?id=384985&showOriginal=true&isReview=true.

**Critical relevance statement:**

This randomized clinical trial carried out in adults undergoing a non-emergent CT scan demonstrates that fasting as a preparation before a contrast-enhanced CT scan should be discontinued and reserved only for certain specific imaging tests.

**Key Points:**

- Despite low osmolar CT contrast media becoming ubiquitous, preparatory fasting is still widely practiced.
- The overall incidence of acute adverse reactions was unchanged after abolishing preparative fasting.
- Traditional preparatory fasting should be discontinued and reserved only for certain specific imaging tests.

**Graphical Abstract:**

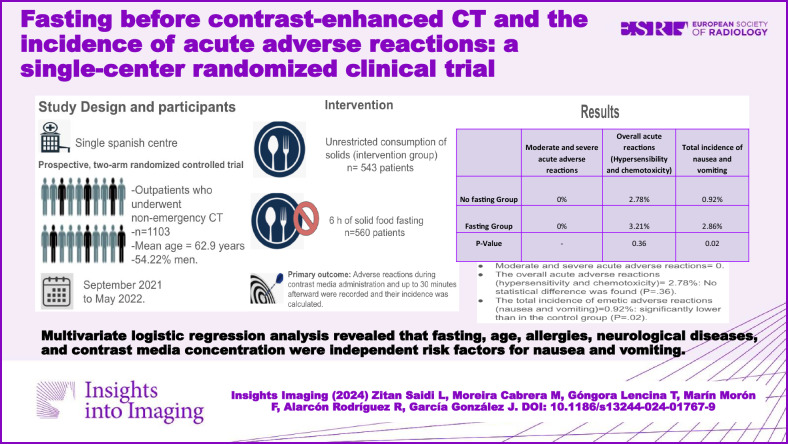

## Introduction

Fasting before contrast-enhanced CT was historically practiced to reduce the risk of nausea and vomiting and protect the patient from pulmonary aspiration of gastrointestinal contents [1–3].

Nausea and vomiting were frequent with the use of high osmolality contrast media (CM) (4.58% and 1.84%, respectively), and gastrointestinal emptying was thought to reduce the risk of these acute adverse reactions (AAR) [4–6]. Therefore, when high osmolality CM was used, preparative fasting before contrast-enhanced CT might be justified. However, after the introduction of low osmolar CM the frequency of emesis has declined markedly (1.04% for nausea and 0.36% for vomiting), and nowadays, nearly all radiological clinics in industrialized countries use only low osmolar CM, which is associated with a lower risk of nausea and vomiting [7–9].

The preparative fasting policies in medical centers worldwide differ significantly, and the policies on the specified fasting periods are considerably variable [7, 9]. From 1994 to the present, several large-scale clinical studies have investigated the risk factors for nausea, vomiting, and aspiration pneumonia after low-osmolality CM exposure. These studies showed that fasting duration was not associated with nausea and vomiting complications and that excessive fasting can elevate the risk of dehydration and CM-induced nephropathy [10–12].

Fasting before a contrast-enhanced CT is no longer recommended by major radiology societies. The latest European Society of Urogenital Radiology guidelines and American College of Radiology guidelines clearly state that fasting is not recommended before routine intravenous CM administration [13, 14]. Nonetheless, fasting before the contrast-enhanced CT examination is still customary in many hospitals worldwide [6, 7].

Thus, this randomized clinical trial aimed to evaluate the effect of eliminating fasting before exposure to low-osmolality CM for a CT scan on the incidence of nausea, vomiting, and other AAR, and to identify associated factors in outpatients undergoing non-emergency contrast-enhanced CT.

## Methods

### Ethical approval

Approved by the local Ethics Committee (Torrecárdenas University Hospital, authorization number: 108/2021). All participants signed a written informed consent form, following all principles and guidelines of the Declaration of Helsinki [15]. The trial was registered on the New Zealand registry as ACTRN12623000071628.

### Study design

This is a single-center, two-arm randomized clinical trial using non-fasting as an intervention.

### Participants

Participants were recruited at Torrecárdenas University Hospital (Andalucia, Spain) from September 2021 to May 2022.

Inclusion criteria: non-hospitalized patients aged 18 years or older, referred for a non-emergency contrast-enhanced CT.

Exclusion criteria*:* incomplete form data, intravenous CM extravasation, antiemetic and prokinetic medication 8–12 h before the examination, altered consciousness, uncontrolled hyperthyroidism, history of allergic reaction or hypersensitivity to iodinated CM, and no written informed consent.

### Randomization

Patients who fulfilled the inclusion criteria were recruited and accepted to participate in the study. Randomization was carried out by a statistician using a computerized random number generator for randomization sequence generation. Simple randomization with a 1:1 allocation ratio was used without stratification.

Experienced nurses and CT technicians in charge notified patients about their assignment arm (fasting or non-fasting); changing groups was not allowed.

Radiologists were blinded to the participants’ allocation until the CT examination.

### Procedures and intervention

Participants were randomly assigned to either at least 6 h of preparative solid food fasting (control group) or to an unrestricted consumption of solids up to the time of CT (intervention group). There were no fluids and medication ingestion restrictions for any case.

Before the examination, the indications and contraindications for all patients were also determined according to the CM application guideline [13] and all patients completed a medical history questionnaire to screen for potential risk of allergy. Consent for CM administration was then obtained.

Two radiology nurses observed the patients during the CM administration and up to 30 min afterward and recorded all the AAR using the same standard sheet.

Because participants were outpatients, a telephone survey was initiated to determine the prevalence of aspiration pneumonia among patients, however, telephone number changes made it impossible to contact all patients. Therefore, the assessment of aspiration pneumonitis and death was performed by reviewing emergency department visits due to acute respiratory symptoms within 96 h after the CT scan.

### CT examination and CM injection protocol

Five 64-slice multidetector CT scans were used (General Electric Medical Systems revolution EVO). Head and neck, chest, abdominopelvic, heart, and vascular studies (angiographic studies of any part of the body) were performed depending on the clinical indication of the exam.

All contrast-enhanced CT examinations were performed with one single type of nonionic CM injected by a high-pressure injector using an 18 G, 20 G, and 22 G needle. The intravenous injection rate ranged from 1 mL/s to 5 mL/s.

The CM used was Ioversol 300 mg, 320 mg, and 350 mg iodine per milligram (Optiray^®^).

The injection doses and rates of CM were selected according to patient age, weight, and the purpose of CT examinations, based on our institutional protocol following the ESUR guidelines [13] and controlled after by the radiologists.

### Data collection instrument

For each patient, we used the same standard record sheet self-designed to register the variables: the date of examination, sociodemographic variables (sex, age, body weight, height, and body mass index), the fasting status and fasting time, the examination sites performed (head and neck, abdomen and pelvis, chest, heart and vascular, and angiographic studies of any part of the body), CM iodine concentration, the volume of CM administered, needle gauge and injection rate, risk factors and underlying diseases (CM-AAR history excluding CM allergy/hypersensitivity, previous history of CM usage, all type of allergies, renal insufficiency, diabetes, hypertension, heart and vascular disease, and neurological, oncological, respiratory, digestive and endocrine disease).

We completed the information on risk factors and underlying diseases from the electronic medical records of the enrolled patients. We categorized the patients by the ordering service; for example, we grouped patients with neurological conditions identified by the Neurology Service. Patients with a low level of consciousness were excluded.

All the AAR (occurrence condition, occurrence time, accompanying symptoms, and outcome of nausea and vomiting) were recorded using the same standard sheet. Variables related to the degree of severity of the AAR were classified according to the European Society of Urogenital Radiology guidelines version 10.0 [13].

### Sample size calculation

We used previously reported incidence of nausea and vomiting following intravenous contrast agent injection [7, 16] as a proxy measure for estimating the sample size required for this study. The sample size for this study was calculated to detect a twofold (RR = 2) increase in rates of nausea from 2.5% to 5%, with a power of 80% and a two-sided significance level of 0.05 for α, between the two groups the sample size to be achieved would be at least 600 individuals, 300 in each study arm. The sample size was reached for the statistical analysis (*n* = 1103).

For each enrolled subject undergoing more than one ambulatory contrast-enhanced CT during the study period, we ensured that only the first observation was collected.

We ensured that only the first observation was collected for each enrolled subject undergoing more than one ambulatory contrast-enhanced CT during the study period.

### Statistical analysis

SPSS Statistics version 26 (IBM Inc., Armonk, NY, USA) was employed for statistical analyses.

Continuous variables were described by mean and standard deviation or median and interquartile interval (or range) according to the normality of data.

Categorical variables were presented as frequencies and percentages.

Shapiro–Wilk test was used to assess the normality of distributions.

Pearson Chi-square test or Fisher’s exact test as appropriate was used to compare binary outcomes. *p* < 0.05 was considered statistically significant.

Comparison of means between groups was performed using the *t*-test or Mann–Whitney test as appropriate.

Various patient factors were evaluated to identify risk factors for nausea and vomiting using univariate and multivariate logistic regression analyses. Variables with high predictive power (Wald test, *p*-value of 0.15) were selected and fed into a final multivariate logistic regression model to identify the risk factors associated with AAR.

## Results

### Sociodemographic and clinical data

**Fig. 1:**
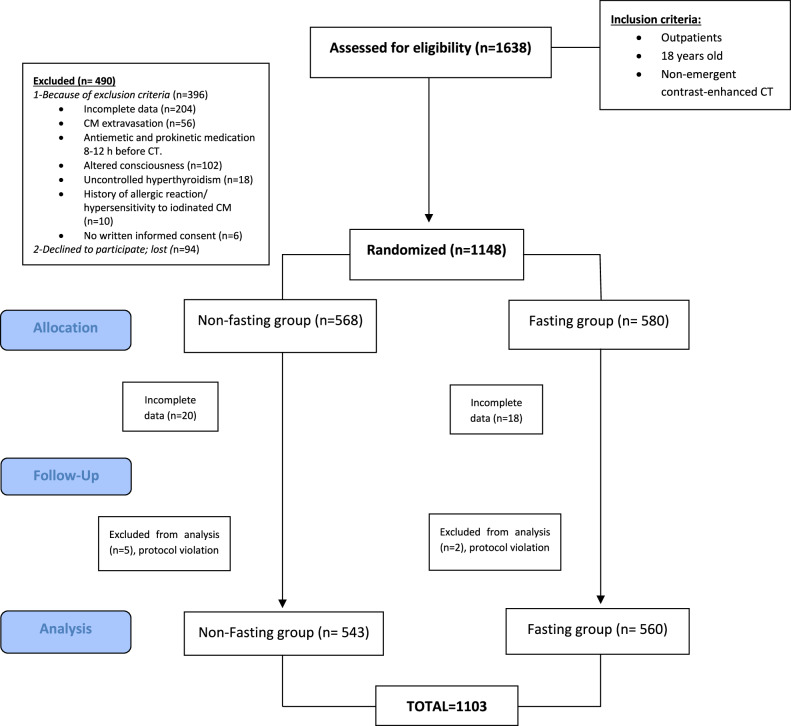
Flow diagram of patients’ enrollment

A total of 1638 participants were recruited, 490 were excluded because of inclusion criteria (*n* = 369) and because of rejection to participate (*n* = 94). A total of 1148 patients were enrolled and randomized. After excluding participants due to incomplete data (*n* = 38) and to protocol violation (patients who finally underwent unenhanced CT or received antiemetic medication before the CT scan, *n* = 7), a total of 1103 patients were evaluated, 560 patients were assigned to the fasting group, and 543 patients to the non-fasting group (Fig. 1).

Patients had a mean age of 62.90 ± 14.80 years and 54.22% were men.

Most participants in both groups underwent abdominopelvic CT (73.40%), with neoplasms as the most frequent underlying disease (33.82%) (Table 1).

**Table 1:** Summary of patient characteristics in both groups

Characteristics	Non-fasting group, (*n* = 543)	Fasting group, (*n* = 560)	Total, (*n* = 1103)	*p* value
Sex				
Male	310 (57.09%)	288 (51.43%)	598 (54.22%)	0.06^A^
Age means (SD), y	62.50 (14.90)	63.40 (14.60)	62.90 (14.80)	0.37^B^
BMI mean (SD), kg/m^2^	26.80 (5.20)	27 (5.20)	27 (5.20)	0.30^B^
Examination sites				
Head and neck	21 (3.87%)	22 (3.93%)	43 (3.90%)	0.96^A^
Abdomen and pelvis	403 (74.21%)	408 (72.86%)	811 (73.52%)	0.66^A^
Chest	281 (51.75%)	283 (50.54%)	564 (51.13%)	0.69^A^
Heart and vascular (angiographic studies of any part of the body)	62 (11.42%)	69 (12.32%)	131 (11.88%)	0.64^A^
Risk factors and underlying diseases				
CM-AAR history (excluding CM allergy/hypersensitivity)	8 (1.47%)	3 (0.54%)	11 (1.00%)	0.12^A^
Previous history of CM usage	356 (65.56%)	406 (72.50%)	762 (69.08%)	0.01*^,^^A^
Allergies	101 (18.60%)	125 (22.32%)	226 (20.49%)	0.13^A^
Asthma	28 (5.16%)	29 (5.18%)	57 (5.17%)	0.99^A^
Diabetes	105 (19.34%)	90 (16.07%)	195 (17.68%)	0.16^A^
Hypertension	201 (37.02%)	212 (37.86%)	413 (37.44%)	0.77^A^
Coronary heart and vascular disease	48 (8.84%)	58 (10.36%)	106 (9.61%)	0.39^A^
Neurological diseases	36 (6.63%)	39 (6.96%)	75 (6.80%)	0.83^A^
Renal insufficiency	16 (2.95%)	21 (3.75%)	37 (3.35%)	0.46^A^
Neoplastic disease	195 (35.91%)	178 (31.79%)	373 (33.82%)	0.15^A^
Respiratory disease	21 (3.87%)	32 (5.71%)	53 (4.81%)	0.15^A^
Digestive disease	70 (12.89%)	91 (16.25%)	161 (14.60%)	0.11^A^
Endocrine disease	15 (2.76%)	18 (3.22%)	33 (2.99%)	0.66^A^
CM concentration				
300	13 (2.39%)	11 (1.96%)	24 (2.18%)	0.88^A^
320	467 (86%)	482 (86.07%)	949 (86.04%)	
350	63 (11.60%)	67 (11.96%)	130 (11.79%)	
CM concentration mean (SD)	323 (10.26)	323.19 (10.27)	323.13 (10.26)	0.73^B^
CM volume mean (SD)	82.28 (11.67)	81.65 (8)	81.96 (12.14)	0.50^B^
Injection rates mean (SD)	3.40 (0.50)	3.30 (0.50)	3.30 (0.50)	0.47^B^

The same patient might have multiple risk factors and underlying diseases simultaneously

*CM* contrast media, *AAR* acute adverse reactions

* Statistically significant

^A^ *p* value was obtained by Pearson’s Chi-squared test

^B^ *p* value obtained by Wilcoxon rank sum test

Most patients had a previous history of CM exposure 762/1103 (69.08%). The mean CM concentration was 323.13 ± 10.26, the mean CM volume was 81.96 ± 12.14, and the mean injection rate was 3.30 (0.50) (Table 1).

### Incidence of AAR after CM administration

The overall AAR (including hypersensitivity reactions and chemotoxicity) incidence was 2.78%. No statistical difference was found in the overall AAR incidence between the fasting group and the non-fasting group (3.21% vs 2.30% *p* = 0.36) (Table 2).

**Table 2:** Incidence of AAR in both groups

	Non-fasting group, (*n* = 543)	Fasting group, (*n* = 560)	Total, (*n* = 1103)	*p* value
Overall AAR excluding heat	12 (2.30%)	18 (3.21%)	30 (2.78%)	0.36^A^
Allergy-like/hypersensitivity				
Mild				
Urticaria	0 (0%)	0 (0%)	0 (0%)	1.0^B^
Pruritus	2 (0.37%)	0 (0%)	2 (0.18%)	0.24^B^
Erythema	1 (0.18%)	1 (0.18%)	2 (0.18%)	1.0^B^
Moderate				
Moderate urticaria	0 (0%)	1 (0.18%)	1 (0.09%)	1.0^B^
Mild bronchospasm	1 (0.18%)	0 (0%)	1 (0.09%)	0.49^B^
Facial edema	0 (0%)	0 (0%)	0 (0%)	1.0^B^
Laryngeal edema	0 (0%)	0 (0%)	0 (0%)	1.0^B^
Severe				
Hypotensive shock	0 (0%)	0 (0%)	0 (0%)	1.0^B^
Respiratory arrest	0 (0%)	0 (0%)	0 (0%)	1.0^B^
Heart attack	0 (0%)	0 (0%)	0 (0%)	1.0^B^
Chemotoxic				
Mild				
Nausea	4 (0.74%)	12 (2.14%)	16 (1.45%)	0.05*^,^^A^
Vomiting	2 (0.37%)	5 (0.89%)	7 (0.63%)	0.45^B^
Heat	402 (74.03%)	430 (76.79%)	832 (75.43%)	0.29^A^
Shaking chills	1 (0.18%)	0 (0%)	1 (0.09%)	0.49^B^
Anxiety	0 (0%)	1 (0.18%)	1 (0.09%)	1.0^B^
Self-limited vasovagal reaction	2 (0.37%)	3 (0.54%)	5 (0.45%)	1.0^B^
Moderate				
Severe vasovagal reaction	0 (0%)	0 (0%)	0 (0%)	1.0^B^
Severe				
Arrhythmias	0 (0%)	0 (0%)	0 (0%)	1.0^B^
Convulsion	0 (0%)	0 (0%)	0 (0%)	1.0^B^
Death	0 (0%)	0 (0%)	0 (0%)	1.0^B^

*CM* contrast media, *AAR* acute adverse reactions

* Statistically significant

^A^ *p* value was obtained by Pearson’s Chi-squared test

^B^ *p* value obtained by Fisher’s exact test for count data

Excluding heat (75.43%), the most common AAR reported by participants in both groups was nausea (1.45%). The total incidence of vomiting in patients was also low 0.63%. The frequency of patients reporting AAR after contrast-enhanced CT scans is shown in Table 2.

Neither vomiting nor nausea caused a patient to be unable to tolerate the examination that occurred in the enrolled patients. No moderate or severe reactions were reported. No aspiration pneumonia or death occurred (Table 2).

The total incidence of nausea was significantly lower in the non-fasting group than in the fasting group (0.74% vs 2.14%, *p* = 0.05).

### The relationship between dietary preparation and the incidence of nausea and vomiting

The total incidence of adverse gastrointestinal symptoms (nausea and vomiting, both together) was comparable between the fasting and non-fasting groups.

We found that the incidence of nausea and vomiting was significantly lower in the non-fasting group than in the fasting group (0.92% vs 2.86%, *p* = 0.02) (Table 3).

**Table 3:** AAR (nausea and vomiting) occurrence in different patient subgroups

Characteristics	Non-nausea and vomiting, (*n* = 1082)	Nausea and vomiting, (*n* = 21)	Total, (*n* = 1103)	*p* value
Fasting	544 (50.28%)	16 (76.19%)	560 (50.77%)	0.02*^,^^A^
Sex				
Male	589 (54.44%)	9 (42.86%)	598 (54.22%)	0.29^A^
Age means (SD), y	63.20 (14.60)	51.50 (16.90)	62.90 (14.80)	0.01*^,^^B^
BMI (mean (SD), kg/m^2^	27 (5.20)	28.80 (8.00)	27 (5.20)	0.50^B^
Examination sites				
Head and neck	43 (3.97%)	0 (0%)	43 (3.90%)	1^C^
Abdomen and pelvis	796 (74.38%)	15 (71.43%)	810 (74.34%)	0.83^A^
Chest	554 (51.20%)	10 (47.62%)	564 (51.13%)	0.74^A^
Heart and vascular (angiographic studies of any part of the body)	125 (11.55%)	6 (28.57%)	131 (11.88%)	0.03*^,^^C^
Risk factors and underlying diseases				
CM-AAR history (excluding CM allergy/hypersensitivity)	11 (1.02%)	0 (0%)	11 (1.00%)	1^C^
Previous history of CM usage	745 (68.85%)	17 (80.95%)	762 (69.08%)	0.23^A^
All kinds of allergies	218 (20.15%)	8 (38.10%)	226 (20.49%)	0.01*^,^^C^
Asthma	55 (5.08%)	2 (9.52%)	57 (5.17%)	0.30^C^
Diabetes	193 (17.84%)	2 (9.52%)	195 (17.68%)	0.56^C^
Hypertension	406 (37.52%)	7 (33.33%)	413 (37.44%)	0.69^A^
Coronary heart and vascular disease	105 (9.70%)	1 (4.76%)	106 (9.61%)	0.71^C^
Neurological diseases	71 (6.56%)	4 (19.05%)	75 (6.80%)	0.05*^,^^C^
Renal insufficiency	37 (3.42%)	0 (0%)	37 (3.35%)	1^C^
Neoplastic disease	368 (34.01%)	5 (23.81%)	373 (33.82%)	0.33^A^
Respiratory disease	53 (4.90%)	0 (0%)	53 (4.81%)	0.62^C^
Digestive disease	159 (14.70%)	2 (9.52%)	161 (14.60%)	0.76^C^
Endocrine disease	33 (3.05%)	0 (0%)	33 (2.99%)	1^C^
CM concentration				
300	24 (2.22%)	0 (0%)	24 (2.18%)	0.08^C^
320	934 (86.32%)	15 (71.43%)	949 (86.04%)	
350	124 (11.46%)	6 (28.57%)	130 (11.79%)	
CM concentration mean (SD)	322.44 (9.48)	328.57 (13.89)	323.13 (10.26)	0.08^B^
CM volume mean (SD)	81.98 (12.50)	80.95 (11.81)	81.96 (12.14)	77^b^
Injection rates mean (SD)	3.30 (0.50)	3.40 (0.50)	3.30 (0.50)	0.74^B^

The same patient might have multiple risk factors and underlying diseases

*CM* contrast media, *AAR* acute adverse reactions

* Statistically significant

^A^ *p* value obtained by Pearson’s Chi-squared test

^B^ *p* value obtained by Wilcoxon rank sum test

^C^ *p* value obtained by Fisher’s exact test for count data

The incidence of nausea and vomiting decreased significantly with age (*p* = 0.01) and there was significantly more incidence of nausea and vomiting in patients with heart and vascular studies (*p* = 0.03), patients with allergies (*p* = 0.05), and patients with neurological diseases (*p* = 0.05) (Table 3).

The two groups (non-nausea/vomiting group and nausea/vomiting group) did not differ regarding the rest of the characteristics: sex, body mass index, CM injection rate (mean), volume, and concentration (the data analysis included evaluating specific volume and concentration with the incidence of adverse reactions), rest of examination parts, or others risk factors and underlying diseases (Table 1). None of the differences was statistically significant (*p* > 0.05).

### Identification of risk factors for AAR

In the univariate regression analysis (Table 2), fasting (*p* = 0.02), age (*p* = 0.01), heart and vascular studies (*p* = 0.03), allergies (*p* = 0.05), and neurological diseases (*p* = 0.05) showed a statistically significant difference between the non-nausea/vomiting group and nausea/vomiting group, and CM concentration (*p* = 0.08) was less than 0.15 of the *p* value.

Among them, in the multivariate logistic regression analysis (Table 4), fasting (OR = 3.35; 95% confidence interval [CI] = 1.27–10.48; *p* = 0.02), age (OR = 0.95; 95% CI = 0.92–0.98; *p* = 0.001), allergies (OR = 2.27; 95% CI = 0.87–5.62; *p* = 0.08), neurological diseases (OR = 4.04; 95% CI = 1.08–12.35; *p* = 0.02) and CM concentration (OR = 2.62; 95% CI = 0.96–6.66; *p* = 0.05) were found to be an independent risk factor for nausea or vomiting.

**Table 4:** Multivariate logistic regression analysis of risk factors for nausea or vomiting

	ORc (95% CI)	*p* value	ORa (95% CI)	*p* value
Fasting	3.16 (1.15–8.70)	0.01	3.35 (1.27–10.48)	0.02
*Age			0.95 (0.92–0.98)	0.01
Allergies	2.43 (1.08–5.95)	0.04	2.27 (0.87–5.62)	0.08
Neurological diseases	5.06 (1.09–10.22)	0.02	4.04 (1.08–12.35)	0.02
*CM concentration			2.62 (0.96–6.66)	0.05

*CI* confidence interval, *ORa* adjusted odd ratio, *ORc* crude odd ratio

* Quantitative variables, ORc not calculable

## Discussion

This study performed in outpatient adults showed that allowing unrestricted intake of solids and liquids before a CT scan was not associated with increased rates of AAR. Our findings are consistent with other studies showing that withholding preparative fasting before contrast-enhanced CT scans is safe and does not increase the risk of AAR [4–7, 9, 16, 17].

Our overall incidence of AAR (2.78%) was higher than that in previous literature (Table 4). However, our data appear more consistent with other prospective studies [3, 5, 7, 11].

Prolonged fasting can amplify the stress response of patients and result in prematurely started catabolism, discomfort, irritation, and uncooperativeness during the imaging examination [18, 19]. Barbosa et al [5] evaluated outpatients at a cancer center and reported that the incidence of some symptoms, such as flushing, dizziness, tingling, tremor, pain at the injection site, tachycardia, and headache was lower in the non-fasting group, even before the administration of the CM.

Furthermore, overnight fasting is potentially hazardous. It causes an increased risk of hypoglycemia [20], dehydration, CM-induced nephropathy, impaired muscular function, nausea [21], blood pressure decrease, and severe shock reactions, especially in elderly malnourished patients [22, 23].

In addition, patients often stop taking routine medication during fasting, which may increase the health risk for patients with chronic diseases [24].

The historical concern about aspiration pneumonia, the prime reason for preparative fasting, has never been validated. According to the study by Sutherland et al [25] fasting longer than 3 h fails to reduce the volume of the gastric content and lowers the pH level, placing patients at increased risk for aspiration pneumonia.

In our study, not a single case of moderate or severe reactions was reported in either arm, and no aspiration pneumonia or death occurred, probably related to the very low frequency associated with low osmolar CM [7–9]. In our findings, nausea/vomiting was infrequent, self-limited, and significantly more frequent in the fasting group than in the non-fasting group. This result is consistent with some studies suggesting that fasting increases nausea and vomiting [4, 5, 10, 11, 26]. In fact, in our study fasting was an independent risk factor for nausea/vomiting.

Therefore, the risk of pulmonary aspiration and secondary pneumonia is more likely to be associated with other factors such as consciousness levels or esophageal lesions (e.g., tracheoesophageal fistula) rather than CM.

Lie et al reported that a high injection rate (≥ 5 mL/s) was a risk factor for AAR in adult patients with underlying diseases and risk factors (asthma, cardiac insufficiency, elderly patients) [27–29]. In our study, the injection rate (mean) was not significantly associated with nausea and vomiting.

However, in our results, the CM concentration was an independent risk factor for nausea/vomiting, a higher concentration had a higher incidence of nausea and vomiting.

Neurological diseases were also independent risk factors for nausea/vomiting. This is likely secondary to the higher iodine concentration used in these patients due to vascular studies (e.g., cerebral arteriography), which is related to a higher incidence of nausea and vomiting. This finding is consistent with previous studies [9, 23].

It is striking that the fasting group had a higher rate of nausea and vomiting, but also had a higher proportion of previous CM use.

The incidence of nausea or vomiting decreased with age, being an independent risk factor for both. Previously, Li et al [17] reported that chemotoxic and allergy-like/hypersensitive reaction incidences decreased with age. The reason for this is unclear, possibly being related to psychological factors, particularly in patients under 29 years [30].

In previous studies [26, 30], the incidence of nausea/vomiting was higher in patients with previous iodine AAR history. In our findings, the incidence of nausea/vomiting was higher in patients with allergies which was also an independent risk factor for nausea/vomiting. This finding may be explained by the fact that patients with a history of adverse reactions to CM and patients with allergies tend to be nervous about CM administration, which may have increased the incidence of adverse reactions.

Non-fasting could be cost-effective since it would reduce waiting time in radiology departments and avoid unnecessary delays or cancellations of examinations.

Patients undergoing contrast-enhanced CT scans should be allowed to unrestricted intake of food and liquids, ensuring a normal metabolic state, improving comfort and cooperation, and reducing the risk of AAR, being recommended only before specific examinations such as oral contrast CT, virtual colonoscopy, CT enterography, and examinations under sedation.

### Limitations

This study was conducted in a single institution and only on adult outpatients referred for a non-emergency scan to obtain a homogeneous sample. Although it limits the generalization of our findings, in our experience emergency and inpatients who do not fast before an enhanced CT hardly suffer adverse reactions.

One type of nonionic CM was tested, but most nonionic CM have a similar composition and are equally unlikely to cause AAR. Even so, our findings cannot be extrapolated to other types of CM.

## Conclusions

Unrestricted food ingestion did not increase the overall incidence of AAR. In our study, preparative fasting increased the incidence of nausea and vomiting. Multivariate logistic regression analysis revealed that fasting, age, allergies, neurological diseases, and CM concentration were independent risk factors for nausea and vomiting.

Since no scientific evidence supports the practice of preparative fasting before contrast-enhanced CT, this practice should be reserved for specific imaging examinations (e.g., oral contrast CT, virtual colonoscopy, CT enterography, and examinations under sedation).

## Data Availability

De-identified individual participant data collected during the trial (date of CT scan, date of birth, age, sex, weight, height, body mass index, assignment arm, intake of antiemetic and prokinetic medication before the examination, allergies history, previous history of CM usage, underlying diseases, anatomic region evaluated, clinical indication, and adverse drug reaction presented) and additional related documents (study protocol, patient information sheet, informed consent, and self-designed sheet to collect the data), will be available on request from the corresponding author after the study is published, with no end date.

## Abbreviations

AAR: Acute adverse reactions
CM: Contrast media

## References

[1] Liu H, Zhao L, Liu J et al (2022) Change the preprocedural fasting policy for contrast-enhanced CT: results of 127,200 cases. Insights Imaging. 10.1186/s13244-022-01173-z 10.1186/s13244-022-01173-z PMC8873329 35201528

[2] McClave SA, Marsano-Obando LS (2022) Preparative fasting orders for medical/surgical interventions and imaging studies: time to review and revise! Curr Gastroenterol Rep. 10.1007/s11894-022-00841-w 10.1007/s11894-022-00841-w 35239128

[3] Kim YS, Yoon SH, Choi YH, Park CM, Lee W, Goo JM (2018) Nausea and vomiting after exposure to non-ionic contrast media: incidence and risk factors focusing on preparatory fasting. Br J Radiol. 10.1259/bjr.20180107 10.1259/bjr.20180107 PMC6221763 29694239

[4] Tsushima Y, Seki Y, Nakajima T et al (2020) The effect of abolishing instructions to fast before contrast-enhanced CT on the incidence of acute adverse reactions. Insights imaging. 10.1186/s13244-020-00918-y 10.1186/s13244-020-00918-y PMC7584708 33095342

[5] Barbosa PNVP, Bitencourt AGV, Tyng CJ et al (2018) Journal club: preparative fasting for contrast-enhanced CT in a cancer center: a new approach. AJR Am J Roentgenol. 10.2214/AJR.17.19061 10.2214/AJR.17.19061 29570378

[6] Neeman Z, Abu Ata M, Touma E et al (2021) Is fasting still necessary prior to contrast-enhanced computed tomography? A randomized clinical study. Eur Radiol. 10.1007/s00330-020-07255-0 10.1007/s00330-020-07255-0 32901302

[7] Ha JY, Choi YH, Cho YJ et al (2020) Incidence and risk factors of nausea and vomiting after exposure to low-osmolality iodinated contrast media in children: a focus on preparative fasting. Korean J Radiol. 10.3348/kjr.2019.0835 10.3348/kjr.2019.0835 PMC7458863 32767861

[8] Suh YJ, Yoon SH, Hong H et al (2019) Acute adverse reactions to nonionic iodinated contrast media: a meta-analysis. Invest Radiol. 10.1097/RLI.0000000000000568 10.1097/RLI.0000000000000568 30998567

[9] Lee BY, Ok JJ, Abdelaziz Elsayed AA, Kim Y, Han DH (2012) Preparative fasting for contrast-enhanced CT: a reconsideration. Radiology. 10.1148/radiol.12111605 10.1148/radiol.12111605 22517959

[10] Oowaki K, Saigusa H, Ojiri H et at (1994) Relationship between oral food intake and nausea caused by intravenous injection of iodinated contrast material. Nihon Igaku Hoshasen Gakkai Zasshi 54:476–479 8028954

[11] Wagner HJ, Evers JP, Hoppe M, Klose KJ (1997) Muss der patient vor intravasaler applikation eines nichtionischen Kontrastmittels nüchtern sein? Ergebnisse einer kontrollierten untersuchung [must the patient fast before intravascular injection of a non-ionic contrast medium? Results of a controlled study]. Rofo. 10.1055/s-2007-1015444 10.1055/s-2007-1015444 9198507

[12] Hwan Seok Yong MD (2020) Preparative fasting before contrast-enhanced computed tomography. J Korean Med Assoc. 10.5124/jkma.2020.63.3.151

[13] European Society of Urogenital Radiology (2018) ESUR guidelines on contrast media ver. 10.0. Available via https://www.esur.org/esur-guidelines-on-contrast-agents. Accessed 16 Aug 2021

[14] American College of Radiology Committee on Drugs and Contrast Media (2022) ACR manual on contrast media. Available via https://www.acr.org/Clinical-Resources/Contrast-Manual. Accessed 6 Jan 2023

[15] World Medical Association (2013) World Medical Association Declaration of Helsinki: ethical principles for medical research involving human subjects. JAMA. 10.1001/jama.2013.281053 10.1001/jama.2013.281053 24141714

[16] Correa-Arruda WS, Vaez IDA, Aguilar-Nascimento JE, Dock-Nascimento DB (2019) Effects of overnight fasting on handgrip strength in inpatients. Einstein. 10.31744/einstein_journal/2019AO4418 10.31744/einstein_journal/2019AO4418 PMC6333214 30652738

[17] Li X, Liu H, Zhao L et al (2018) The effect of preparative solid food status on the occurrence of nausea, vomiting, and aspiration symptoms in enhanced CT examination: a prospective observational study. Br J Radiol 91:20180198. 10.1259/bjr.20180198 10.1259/bjr.20180198 PMC6350464 29906236

[18] Park JH, Rhee JE, Kim KS, Shin JH (2008) Is the six-hour fasting before abdominal computed tomography necessary to prevent gastrointestinal adverse events in patients with abdominal pain? J Korean Soc Emerg Med 19:333–338

[19] Carey SK, Conchin S, Bloomfield-Stone S (2015) A qualitative study into the impact of fasting within a large tertiary hospital in Australia--the patients’ perspective. J Clin Nurs. 10.1111/jocn.12847 10.1111/jocn.12847 25959390

[20] Sorita A, Thongprayoon C, Ahmed A et al (2015) Frequency and appropriateness of fasting orders in the hospital. Mayo Clin Proc. 10.1016/j.mayocp.2015.07.013 10.1016/j.mayocp.2015.07.013 26355400

[21] Morcos SK, Thomsen HS (2001) Adverse reactions to iodinated contrast media. Eur Radiol. 10.1007/s003300000729 10.1007/s003300000729 11471623

[22] Wickerham AL, Schultz EJ, Lewine EB (2017) Nil per Os orders for imaging: a teachable moment. JAMA Intern Med. 10.1001/jamainternmed.2017.3943 10.1001/jamainternmed.2017.3943 28975225

[23] Nagamoto M, Gomi T, Terada H, Terada S, Kohda E (2006) Evaluation of the acute adverse reaction of contrast medium with high and moderate iodine concentration in patients undergoing computed tomography. Radiat Med. 10.1007/s11604-006-0087-1 10.1007/s11604-006-0087-1 17186321

[24] Liu H, Liu Y, Zhao L, Xue Li, Zhang W (2021) Preprocedural fasting for contrast-enhanced CT: when experience meets evidence. Insights Imaging. 10.1186/s13244-021-01131-1 10.1186/s13244-021-01131-1 PMC8643287 34865183

[25] Hamid T, Aleem Q, Lau Y et al (2014) Pre-procedural fasting for coronary interventions: Is it time to change practice? Heart. 10.1136/heartjnl-2013-305289 10.1136/heartjnl-2013-305289 24522621

[26] Li X, Liu H, Zhao L et al (2017) Clinical observation of adverse drug reactions to non-ionic iodinated contrast media in populations with underlying diseases and risk factors. Br J Radiol 90:20160729. 10.1259/bjr.20160729 10.1259/bjr.20160729 PMC5685114 27928926

[27] Clauss MW (1999) What is the place of fasting and dehydration before contrast media administration? In: Dawson P (ed) Contrast media in practice: question and answers, 2nd ed. New York, NY: Springer-Verlag, p 118–119

[28] Sutherland AD, Maltby JR, Sale JP, Reid CR (1987) The effect of preoperative oral fluid and ranitidine on gastric fluid volume and pH. Can J Anaesth. 10.1007/BF03015327 10.1007/BF03015327 3829296

[29] Bottinor W, Polkampally P, Jovin I (2013) Adverse reactions to iodinated contrast media. Int J Angiol. 10.1055/s-0033-1348885 10.1055/s-0033-1348885 PMC3770975 24436602

[30] Gomi T, Nagamoto M, Hasegawa M et al (2010) Are there any differences in acute adverse reactions among five low-osmolar non-ionic iodinated contrast media? Eur Radiol. 10.1007/s00330-009-1698-6 10.1007/s00330-009-1698-6 20033176

